# A True Fibroma in the Hard Palate: A Report of a Rare Case

**DOI:** 10.7759/cureus.57182

**Published:** 2024-03-29

**Authors:** Lakshmi Narayanan S, Manoj Margabandhu, Kennedy Babu, Gandhimadhi D, Soorya K.V

**Affiliations:** 1 Department of Periodontology, Mahatma Gandhi Postgraduate Institute of Dental Sciences, Pondicherry, IND

**Keywords:** oral mucosa, benign neoplasm, hard palate, fibroma, diode laser

## Abstract

The incidence of benign neoplasms is common in the oral cavity. Provisional diagnosis does not accurately identify rare neoplasms. With differential diagnosis of similar lesions and confirmation by histopathology, rare lesions can be identified. This case report also ended up to be a rare lesion of true fibroma in the palate through a histopathology report. Hence, the correlation of clinical findings to the confirmation with histopathology leads to a definitive diagnosis of uncommon lesions.

## Introduction

Fibroma is a benign neoplasm characterized by the excessive proliferation of fibroblasts with large amounts of collagen [1]. The etiology of fibroma has been reported to the chronic trauma from poorly fitting denture, calculus, overhanging restoration, sharp fragments of fractured or attrited teeth, and injury from self-bite. They are either pedunculated or sessile growths on any surface of the oral mucosa. Most of the lesions are small and less than 1 cm in diameter. Most of the fibroma occurring are not true of its nature. It is generally a reactive lesion in response to external injury or irritation [2]. Only those cases of fibroma that are not caused due to the above-mentioned factors and inherently a proliferative lesion on its own come under the true fibroma category [3]. This case report is unique in that way. Also, an additional uniqueness is that the order of presentation of fibroma in the oral cavity according to the most common sites includes the gingiva, buccal mucosa, and lips, followed by the palate and tongue as the least. Barker and Lucas were the pioneers who elaborated on fibrous overgrowths of oral mucosa [4]. Since then, only a few cases have been reported in the literature [5,6].

## Case presentation

Patient information

A male patient 51 years of age reported to the periodontology department with the chief complaint of swelling in the palate for the past three months. The swelling was initially smaller in size and had progressively increased with time to attain the present size. There was no associated pain, but the patient had discomfort because it interfered with chewing and speech. Medical history was non-contributory. The patient had a history of smoking two cigarettes per day for the past 20 years.

Clinical findings

**Figure 1:**
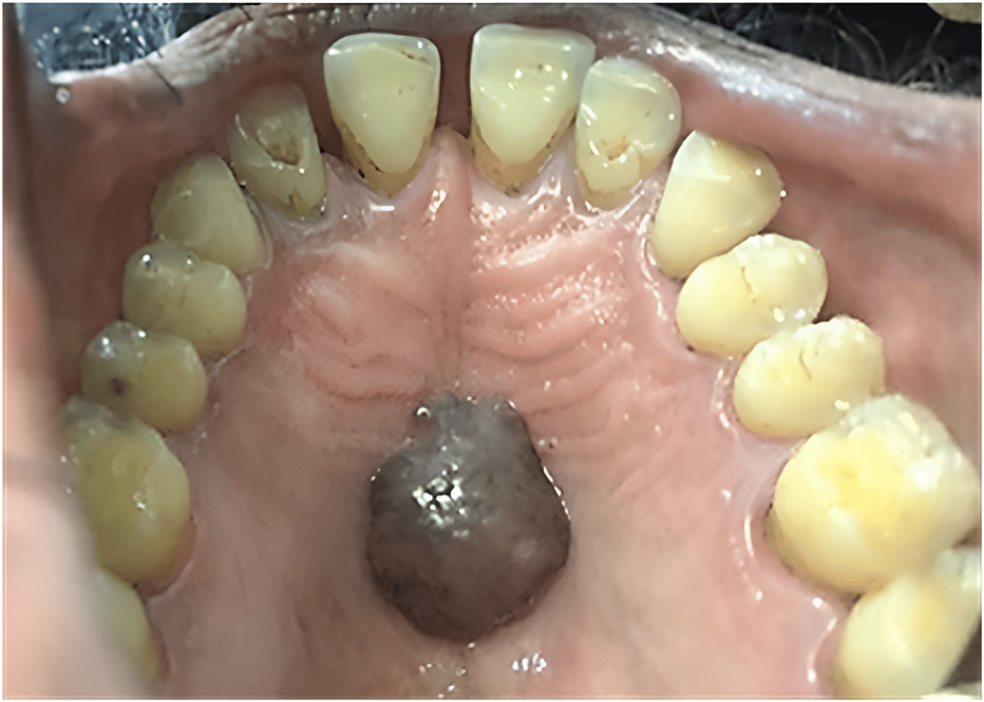
Preoperative picture showing exophytic lesion of size 2×2.5×1 cm in the centre of the hard palate.

On extraoral examination, no gross abnormality was detected. On intraoral examination, an exophytic lesion was present on the centre of the palate which was brown in colour. The lesion was equidistant from the maxillary posteriors on either side in the centre of the palate. It measured about 2×2.5×1 cm in size, and there were no surface changes (Figure 1).

On palpation, findings on inspection were confirmed. The lesion was non-tender and pedunculated. It was firm and non-fluctuant and did not blanch under pressure.

Diagnostic assessment

**Figure 2:**
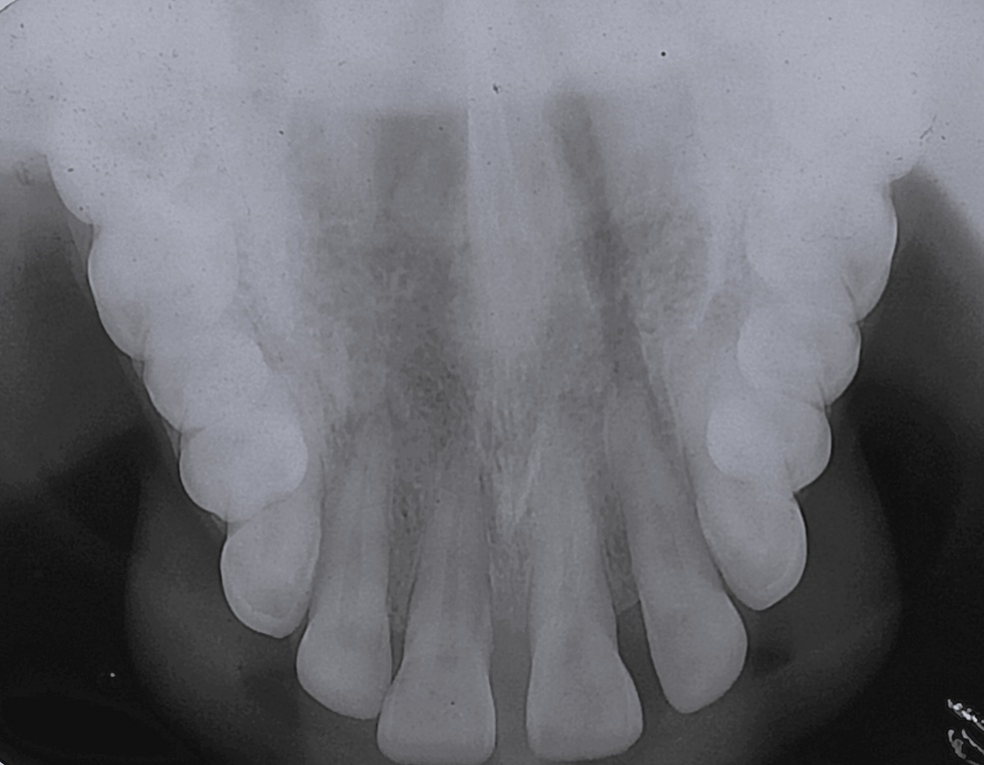
Preoperative occlusal radiograph did not show any pathological bony changes.

The patient was subjected to routine blood investigations and the values were within normal limits. The occlusal radiograph revealed the absence of any pathological bony changes (Figure 2).

Correlating the patient's history and clinical findings, the differential diagnosis included hemangioma, melanoma, peripheral ossifying fibroma, benign minor salivary gland tumor, and palatal tori.

Therapeutic intervention

**Figure 3:**
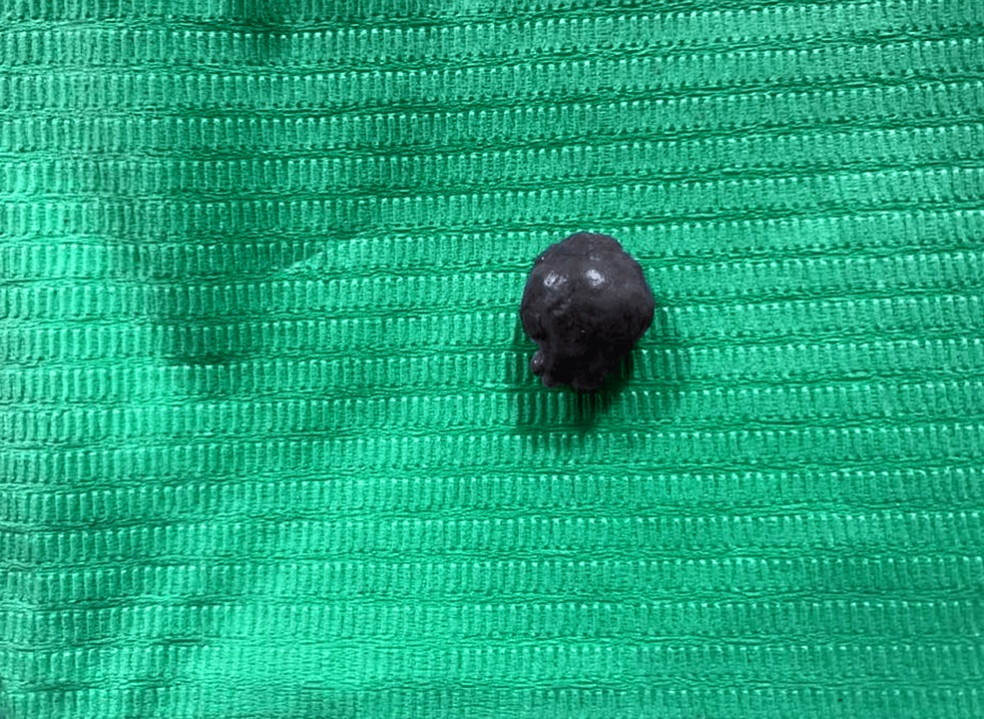
Specimen excised using a diode laser to be sent for histological examination.

The lesion was excised completely under local anaesthesia using a diode laser with a wavelength of 980 nm, a power of 2.5 watts, and continuous contact mode (Figure 3).

**Figure 4:**
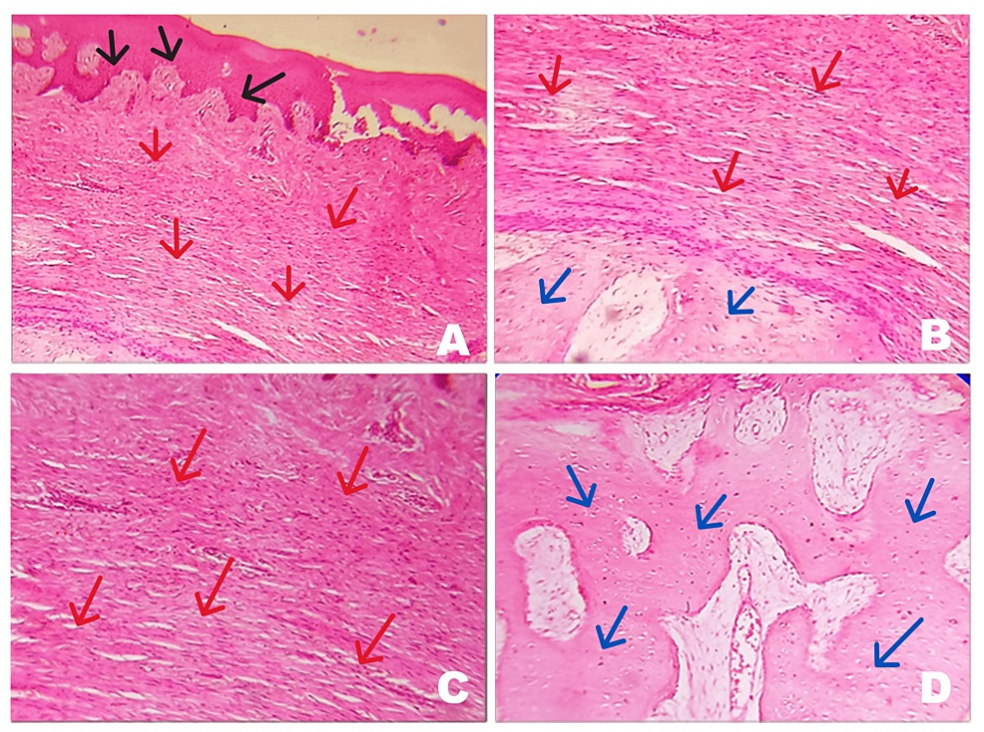
A) The H&E-stained section shows both hard and soft tissue. The section exhibits parakeratinized epithelium of normal thickness (black arrow) and condensed fibrous connective tissue (red arrow) (4×). B) The fibrous structure (red arrow) is interspersed with the ossified tissue (blue arrow) (10×). C) The fibrous connective tissues which in most of the areas are avascular in nature (red arrow) (10×). D) The central part of the lesion is basically made up of ossified tissue formation (blue arrow) (10×).

The tissue fixed in 10% neutral buffered formalin was sent for histological examination in order to establish a confirmatory diagnosis. The histopathological H&E stains revealed that the given section consisted of both soft and hard tissue. The central part of the lesion was basically made of ossified tissue interspersed with a fibrous structure. The whole lesion was covered by fibrous connective tissues, which in most of the areas were avascular in nature. The lesion was well condensed by a fibrous capsule. The overlying epithelium was of normal thickness and covered by keratinized areas (Figure 4A, 4B, 4C, 4D).

The lesion was diagnosed as a long-standing fibroma with ossification changes.

Follow-up and outcome

**Figure 5:**
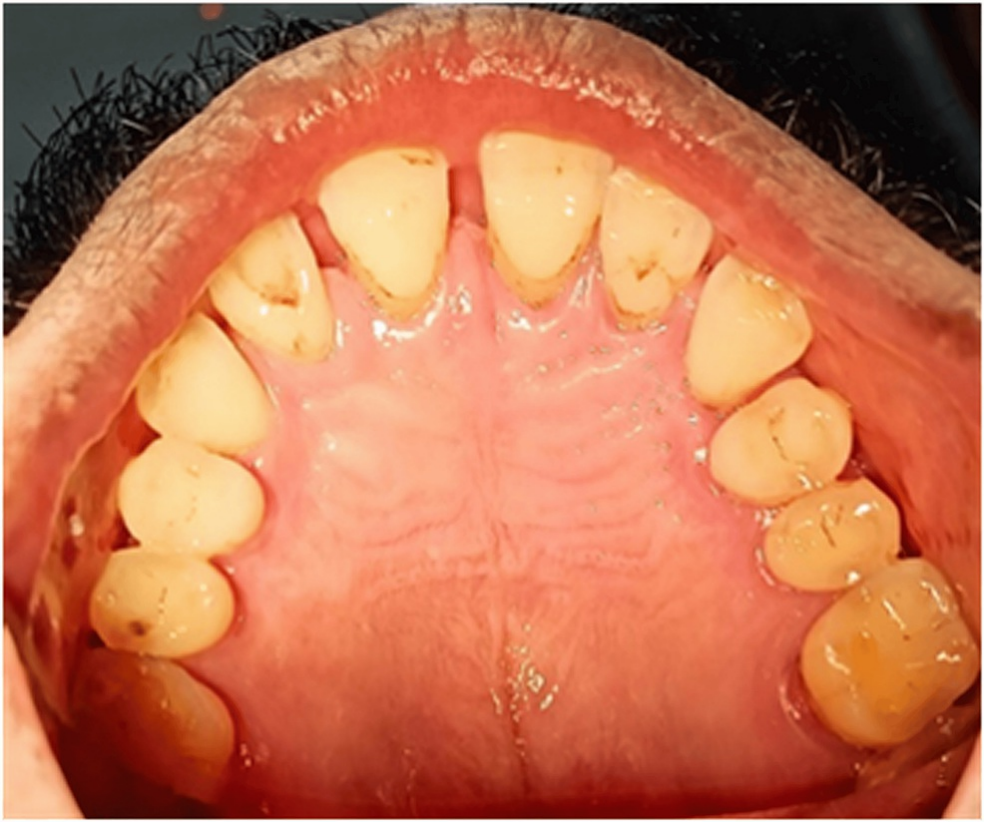
Two months postoperative picture showing satisfactory healing.

The patient was recalled for immediate postoperative and two-month follow-up. The operated site showed satisfactory healing (Figure 5).

## Discussion

The clinical features pointed out a differential diagnosis that was completely different from the histopathological results which suggested a long-standing fibroma with ossification changes. Due to the rarity of occurrence of fibroma reported in the palate, the differential diagnosis did not include the true fibroma. According to Barker and Lucas, the presentation of true fibroma in the palate was rare. In a study of 650 specimens of localized fibrous growths, they concluded that the lesion usually occurred with a higher incidence in females compared to males [4]. However, the cases reported by Jain and Chnadan et al. have shown incidence in male patients [5,6].

According to Burkitt, the average size of a fibroma is usually less than 1-1.5 cm in diameter. Conversely, this case had an average size of 2×2.5 cm in diameter [7]. However, this was analogous to the case reported by Jain who presented a case of fibroma in the palate measuring 5×4 cm. Also, the radiograph did not show any bony changes in both cases [5].

Moving on to histopathology, Barker and Lucas demonstrated two patterns of fibres depending on the location of the fibroma and the existence of trauma. One is the radiating type, seen in mobile areas exposed to trauma as reported by Cohen [8], and the other one is the circular type evident in immobile areas like the palate without trauma [4]. This is in harmony with the histopathology in this case where we could see a circular pattern of arrangement of fibres with the central ossifying tissue suggestive of true fibroma in the palate without trauma. This is also in line with the cases reported by Chnadan et al. and Khan and Gaikwad [6,9].

Laser-assisted surgical excision was planned in order to prevent excessive intraoperative bleeding, for faster healing with minimal scar tissue formation, and for decreased postoperative pain. Chances of post-surgical infection will be less because of the bactericidal effect of laser compared to conventional procedures [10,11].

Hence, the primary take-away lesson from this case report is that, though variations in etiology, sex predilection, clinical location, and presentation were present in this case, one should not always fail to include the rarity in diagnosis as reported by a few authors earlier. Even when the local factor of irritation or trauma was absent, the inherent quality of true fibroma has to be understood and kept in mind while establishing the diagnosis of a benign oral lesion. This patient had a history of smoking cigarettes for the past 20 years. The possibility of palatal tissue response to smoking should be kept in mind in order to prevent recurrence. Hence, the patient has to be subjected to tobacco cessation counseling to stop the habit and avoid recurrence in the future.

## Conclusions

Fibromas are less common in the region of the hard palate. In order to eliminate the most common palatal swellings like tori, the lesion was subjected to radiographic investigations. Absences of bony changes excluded the same. Also, histopathology of the lesion ruled out the possibility of melanoma, hemangioma, and other minor salivary gland tumors making the diagnosis confirmative to be fibroma. Hence, the rarest occurrence of these fibromas in the palate should be considered during the differential diagnosis of further cases in the future.
